# Late Relapse of Previous Pulmonary Cryptococcosis With Symptoms Resembling Cerebral Infarction: A Case Report

**DOI:** 10.1155/2024/3905985

**Published:** 2024-10-04

**Authors:** Anatoli Pinchuk, Gernot Geginat, Volker Rickerts, Belal Neyazi, Klaus Peter Stein, Christian Mawrin, I. Erol Sandalcioglu, Ali Rashidi

**Affiliations:** ^1^Department of Neurosurgery, Otto-Von-Guericke-University Magdeburg, Magdeburg, Germany; ^2^Department of Medical Microbiology and Hospital Hygiene, Otto-Von-Guericke-University, Magdeburg, Germany; ^3^Department of Infectious Diseases, Unit for Mycotic and Parasitic Agents and Mycobacteria, Robert Koch-Institute, Berlin, Germany; ^4^Department of Neuropathology, Otto-Von-Guericke-University, Magdeburg, Germany

**Keywords:** central nervous system, cerebral infarction, *Cryptococcus neoformans*, meningoencephalitis

## Abstract

Cryptococcosis, an infection caused by *Cryptococcus neoformans and Cryptococcus gattii*, predominantly targets the central nervous system (CNS) in patients with AIDS but is not limited to this group. The disease can also occur in individuals with various immunosuppressive conditions, frequently involving the brain or lungs. Cryptococcal meningitis (CM) is the most common form of fungal meningoencephalitis, leading to intracerebral infections, cerebral infarction, or hydrocephalus. The clinical presentation of CM is nonspecific, and imaging features can vary significantly. This case report presents a patient with cerebral infarction, who was HIV-negative but had been on long-term cortisone therapy. Notably, the patient had a history of pulmonary cryptococcosis 15 years prior to cerebral involvement. When initially at our clinic, histology and culture results from brain biopsies were negative and the earlier pulmonary cryptococcosis history was unknown. Subsequently, cryptococcal antigen was detected in both serum and cerebrospinal fluid (CSF), and *C. neoformans* was cultivated from CSF. This case highlights the critical importance of maintaining a high index of suspicion for CM, particularly in patients with a history of previous cryptococcal infections, and it also demonstrates the possibility of false-negative brain biopsy results due to secondary vascular events associated with CM.

## 1. Introduction

Cerebral cryptococcal infection most commonly manifests as meningoencephalitis, and the coexisting central nervous system (CNS) and lung infections are prevalent in cases of AIDS-associated cryptococcal meningoencephalitis, with CNS symptoms typically taking precedence in the clinical presentation [1]. While CNS cryptococcosis is traditionally associated with HIV infection, recent observations in higher income countries indicate an increasing recognition of the infection in individuals without HIV/AIDS [2, 3].

Several prior studies have delved into the clinical epidemiology and outcomes of cryptococcosis in non-HIV individuals. The primary risk groups encompass patients with immunodeficiency, rheumatic diseases, diabetes mellitus, malignancy, and chronic organ diseases affecting the liver, lungs, or kidneys [4, 5]. It is noteworthy that cryptococcosis can also occur in immunologically competent hosts, and in individuals with normal immune function, approximately one-third of affected patients exhibit asymptomatic isolated pulmonary infection [1].

Cryptococcal meningitis (CM) clinically manifests as either a chronic or subacute course. Irrespective of the underlying condition, the majority of patients exhibit clinical features consistent with subacute meningitis, presenting with symptoms such as headache, fever, and vomiting. However, in non-HIV patients, the diagnosis might be delayed due to the low clinical suspicion, absence of fever, and the insidious onset of symptoms. In cases not associated with HIV, confusion (altered mental status), seizures, and focal neurological deficits are the primary clinical features attributed to CM [6].

The pathogenesis of CM remains incompletely understood. One of the crucial steps in the development of the disease is the spread of the fungi and their penetration into the brain by crossing the blood–brain barrier to the CNS by transcytosis, paracellular crossing, or a Trojan horse mechanism after phagocytosis of *Cryptococcus neoformans* by phagocytes [7, 8]. In the brain, an alternative route for the infection's spread may be provided by the perivascular space. Magnetic resonance imaging (MRI) scans in human infections reveal dilated perivascular spaces, known as Virchow–Robin spaces, in patients diagnosed with CM [9].

Tests for fungal antigens facilitate the detection of such antigens in both serum and cerebrospinal fluid (CSF). A cytologically and serologically inconspicuous CSF finding, however, does not definitively exclude the possibility of cryptococcosis. In cases where there is a reasonable suspicion, it is advisable to supplement such findings with an antigen screening in the serum [10, 11].

Signs typical for CM in AIDS patients include dilated Virchow–Robin spaces, pseudocysts, cryptococcoma, and leptomeningeal enhancement [12]. These are characterized by high fungal load and a lack of T-cell response. Compared to AIDS patients, imaging findings in non-AIDS patients are often less pronounced and vascular complications such as vasculitis and infarctions may complicate clinical and image diagnosis and make microbiological diagnostic testing mandatory.

Complications arising from CM encompass increased intracranial pressure and cerebral infarction, necessitating special management strategies such as serial spinal taps or shunt insertion [13]. Infarction of cryptococcomas may occur, potentially linked to vasculitis or local pressure effects, with MRI-based studies revealing an incidence rate of infarction ranging from 18% to 44% [14–16].

In summary, the clinical picture of CM is often unspecific. In a large proportion of patients, this infection is misdiagnosed and/or the diagnosis is delayed. Here, we present a case in which CM was known to be a predisposing factor and yet was initially misdiagnosed as a cerebral infarction.

## 2. Case Presentation

A 69-year-old male patient was initially transferred from a rehabilitation clinic due to a decrease in alertness. In July 2019, the patient had been diagnosed with acute territorial infarction in the right mediastinal region at an external clinic and was subsequently referred to a rehabilitation clinic for poststroke treatment. At the time of admission at the external clinic, the patient exhibited reduced alertness, prompting the ordering of a contrast-enhanced MRI (cMRI) scan. In the external hospital stay, there was a progressive deterioration in his overall condition, leading to further diagnostic investigations. A computed tomography (CT) scan of the thorax and abdomen did not reveal any significant abnormalities. However, the cMRI with contrast demonstrated a contrast-enhancing lesion in the right parietal region accompanied by notable edema without typical signs of CM. In the medical history, it was noteworthy that the patient had been receiving corticosteroid (dexamethasone 10 mg/daily) for chronic obstructive pulmonary disease (COPD) for several years. Subsequently, the patient's cardiopulmonary status further declined, and there was a significant increase in inflammatory markers (leukocytes 10,14 Gpt/l, C-reactive protein (CRP) 210,5 mg/L, and procalcitonin (PCT) 0.78 ng/mL). The CSF analysis at the external clinic revealed an elevated cell count (211^∗^10^6^ per liter) with 3% neutrophils, 80% lymphocytes, 14% monocytes, total protein 1601 mg/L, albumin 947 mg/L, Q albumin (CSF/serum) 38.8, Q IgG 36.6, Q IgA 28.4, Q IgM 9.2, glucose 5.4 mmol/L, and lactate 3.97 mmol/L (Table 1) indicating a pronounced disorder of the blood–brain barrier with possibly autochthonous intrathecal IgG and IgA synthesis and leukocytosis with lympho-monocytic cell picture and artificial blood admixture.

Empirical antibacterial and antiviral therapy was initiated with ceftriaxone, metronidazole, and acyclovir and was later switched to flucloxacillin and metronidazole. At that time, no bacterial, viral, or fungal infection was detected in the serum or CSF. The diagnostic panel comprised varicella zoster virus IgG, rubella virus IgG, measles virus IgG, *Borrelia burgdorferi* IgG/IgM, *Echinococcus* spp IgG, *Toxoplasma gondii* IgG in serum and CSF and *Mycobacterium tuberculosis* PCR, herpes simplex virus 1 PCR, herpes simplex virus 2 PCR, varicella zoster virus PCR, human cytomegalovirus PCR, Epstein–Barr virus PCR, *Tropheryma whipplei* PCR, *Aspergillus* spp PCR, *Candida* spp PCR, and *C*. *neoformans* PCR in CSF. No test for *C. neoformans* antigen and no fungal culture for *C. neoformans* were performed at that time. Despite bronchoscopy and chest CT revealing no evidence of pneumonia, the patient's condition worsened, requiring the use of catecholamines. Subsequently, he was intubated for airway protection. In addition, the patient developed absolute tachyarrhythmia, with a heart rate reaching up to 190 beats per minute, leading to the initiation of amiodarone therapy.

**Figure 1:**
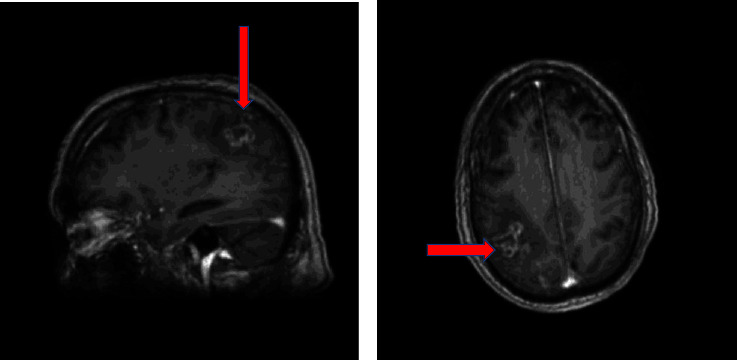
(a, b) MRI of the brain with contrast medium shows a non–space-occupying lesion in the right parietal region with perifocal edema in the sagittal and axial views in the cMRI images without typical signs of CM (red arrow).

The patient was later transferred to our clinic due to an unclear right parietal mass, necessitating further histological examination. Upon admission, the patient was intubated, ventilated, analgosedated with present brainstem reflexes. In the subsequent course, the patient opened his eyes and exhibited minimal movement in the upper and lower extremities. The cMRI revealed a diffuse contrast-enhancing lesion on the right parietal side with significant edema, prompting consideration of various differential diagnoses, including infarction, hemorrhage, brain tumor, or infection (Figure 1).

**Figure 2:**
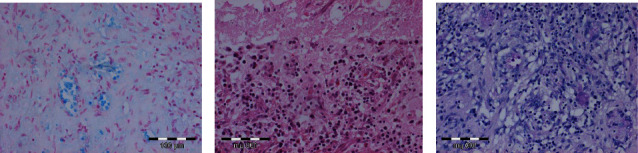
Histopathologic analysis (a–c) of brain sections stained with PAS of the affected brain right parietal shown in the MRI images in Figure 1. Using PAS staining, no fungal elements were detected. The sections show freshly ischemically damaged, reactive-altered tissues with secondary inflammatory changes.

After a thorough evaluation, an open sampling procedure was conducted. Intraoperatively, the situation exhibited characteristics indicative of cerebral infarction. Following the operation, the patient was admitted to our intensive care unit for further therapy. Histological examination showed freshly ischemic portions of damaged CNS tissues without evidence of a tumor. In addition, no signs of cryptococcosis were observed (Figure 2).

The fungal and bacterial culture of the brain biopsy yielded negative results (Table 1). Specifically for the detection of cryptococci, the damaged CNS tissue was cultured for 28 days at 30°C and 37°C on Sabouraud glucose selective agar (Oxoid, Wesel, Germany), supplemented with chloramphenicol and gentamycin. In addition, it was inoculated into Sabouraud bouillon (Biomerieux, Nürtingen, Germany), which was incubated without shaking for 28 days at 30°C and 37°C, respectively. As the CSF data from the previous clinic were only 5 days old, no further CSF diagnostic tests were performed at this time.

Following the diagnosis of cerebral infarction and the absence of bacteria, viruses, or fungi in the biopsy, the initial antibiotic therapy was discontinued. Transesophageal echocardiography (TEE) revealed no evidence of endocarditis.

Persistent pleural effusions on both sides did not improve under conservative management with a negative fluid balance. A pleural puncture was performed, although no microbiology testing was conducted on the pleural fluid. In the case of prolonged weaning, a percutaneous dilatation tracheotomy was performed.

Antibiotic therapy with piperacillin–tazobactam was initiated as inflammatory parameters and fever increased. Radiologically, aspiration pneumonia was suspected. Vancomycin was added due to persistent inflammatory parameters, and in the absence of a specific pathogen, the antibiotic therapy was switched to meropenem plus caspofungin. While leukocyte counts normalized, CRP remained elevated.

As the patient improved clinically, he was transferred to a rehabilitation clinic for further treatment. Three weeks after the transfer of the patient, a lumbar puncture was performed again, and cryptococcus antigen (IMMY cryptococcal antigen lateral flow assay, Immuno-Mycologics Inc., Norman, OK, USA) was detected in the CSF (titer 1:2560) and serum (titer 1:320). *Cryptococcus neoformans* var. *grubii* was cultured from CSF. Molecular typing using the standard seven gene multilocus sequence typing protocol identified sequence type (ST) 2 [17]. This ST is frequently cultivated from patients with cryptococcosis in Europe and can be cultivated from environmental sources including trees [18]. After the confirmed diagnosis of cerebral cryptococcosis, a specific antifungal therapy with liposomal amphotericin B plus flucytosine was initiated. The patient's condition initially remained stable under treatment. However, later, the patient developed a severe septic shock and CT images showed massive brain edema. Due to the patient's very poor general condition and in respect to the patient's will, no further life-sustaining intensive care was performed and the patient died 1 month after discharge from our hospital.

In the context of the postmortem scientific workup of the case, it was recognized that the patient had a previous infection with *C. neoformans* approximately 15 years ago. At that time, because of a peripheral pulmonary nodule in the right lower lobe of the lungs, an exploratory thoracotomy with an atypical wedge resection was performed and a histological examination of the surgical specimens revealed a granulomatous inflammation of the lungs due to cryptococcosis. Remarkably, in the old patient file, no specific antifungal therapy had been documented.

Postmortem the *Cryptococcus* isolate of the patient was typed as *C. neoformans* var. *grubii* by the German reference laboratory for cryptococci. The isolate did not show sufficient growth in various microdilution and agar diffusion tests to determine the minimum inhibitory concentration, which is why an indicative resistance test was carried out using the ellipsometry test on potato water agar. The minimum inhibitory concentrations for amphotericin B was 2 *μ*g/mL (resistant), for 5-fluorocytosine 0.25 *μ*g/mL (sensitive), for fluconazole 12 *μ*g/mL (resistant), and for voriconazole 0.094 *μ*g/mL (sensitive). Resistance of cryptococci against amphotericin B is rare [19]. As the resistance breakpoint for amphotericin B is > 1 *μ*g/mL according to the European Committee on Antimicrobial Susceptibility Testing (EUCAST), the slightly elevated minimal inhibitory concentration of 2 *μ*g/mL is likely a laboratory artifact due to the poor growth of the isolate on artificial media [17].

## 3. Discussion

In this presented case study, we present an immunocompromised patient with subacute CM that was initially misdiagnosed as a cranial mass and cerebral infarction. At the time of initial presentation, the patient's prior medical history of pulmonary cryptococcosis, which had occurred 15 years earlier, was unknown. The patient had been receiving long-term corticosteroid therapy for several years as part of COPD treatment and also had a diagnosis of diabetes mellitus. The primary predisposing factor for cranial CM was likely previously undisclosed history of pulmonary cryptococcosis, which had occurred 15 years prior to the initial presentation.

The clinical manifestations of CM are known for their variability, and the pathophysiology of the ensuing neurological sequelae is intricate [20]. While relapses of CM are not uncommon [21–25], the special feature of our case is that an initial episode of cryptococcosis had happened at a different anatomical site in the lungs 15 years ago in a patient without a known severe immune deficiency. Because we could not compare the current *C. neoformans* isolate with the previous pulmonary isolate, however, the possibility of reinfection cannot be entirely ruled out.

Throughout the course of the disease, our patient did not exhibit the classical acute symptoms associated with CM. The predominant manifestation of CM was a subtle disturbance of consciousness. As previously documented, a reduction in vigilance is recognized as one of the clinical presentations of CM, believed to stem from increased intracranial pressure, a condition that may persist for days or even weeks. In addition, the absence of fever initially precluded suspicion of an infection. Other atypical symptoms observed in our patient included weakness, difficulty walking, and psychotic manifestations.

Despite the presence of a high cell count in the CSF in our patient, the histologic examination of intraoperative materials at that time did not raise suspicions of a cerebral infection and the PCR test performed was negative. Therefore, initially, no specific cryptococcal lateral flow antigen tests were performed [10, 11]. We could not retest the patients' CSF or liquor after the CM diagnosis was made because no material was left. Despite the specificity of the *C. neoformans* PCR being 96%–99%, the sensitivity of the PCR is only 50%–96% compared to the lateral flow assay which in CSF has a sensitivity and specificity of 99%–100% [26].

Imaging changes associated with CM can often be nonspecific and challenging for clinicians to discern. Studies focusing on neuroimaging findings in immunocompetent patients are also limited. An experimental study [24] demonstrated that the primary event in CM involves parenchymal invasion following the penetration of cryptococci across the blood–brain barrier, particularly at the level of cortical capillaries surrounded by the Virchow–Robin space. This observation provides a potential explanation for the brain lesions, including ischemic events, observed in our patient. Intraparenchymal cryptococcomas are mass-like lesions that can mimic a brain tumor or cerebral infarction, mirroring the presentation in our patient (Figure 1).

While CM rarely directly leads to cerebral infarction [21], acute and subacute cerebral infarction may occur in the course of the infection and may involve both the anterior and posterior cerebrovascular territories. The literature describes various types of CM-related cerebrovascular events, encompassing vasculitis, vasospasm, and thrombosis, all of which may contribute to the occurrence of cerebral infarction in CM patients [22, 23].

Because cryptococcomas are infrequent in *C. neoformans* var*. grubii* and brain histology was negative for fungal elements, an alternative explanation would be that CM in this case was a secondary complication after a primary vascular event.

The current case suggests that subarachnoid vein occlusion due to inflammatory fibrosis might be a plausible mechanism for the observed cerebral infarction in CM. In our patient, a cranial MRI examination suggested an acute brain lesion and only a subsequent positive cryptococcal antigen test in both serum and CSF provided conclusive evidence that the brain lesion was indeed attributable to the patient's *C. neoformans* infection.

## 4. Conclusion

We present a case of CM mimicking cerebral infarction, 15 years after untreated pulmonary cryptococcosis in a patient with extended corticosteroid therapy for COPD but without being otherwise severely immunosuppressed. Our patient exhibited a notable decrease in vigilance and absence of fever during the initial phases of the disease. The initial brain biopsy failed to establish the diagnosis of CM that was subsequently established by positive cryptococcal antigen tests from serum and CSF as well as culture of *C. neoformans* from CSF. This case underscores the importance of taking a proper clinical history and a critical importance of maintaining a high index of suspicion for CM, particularly in individuals with a history of prior cryptococcal infection.

## Figures and Tables

**Table 1:** Initial laboratory test results from CSF and intraoperative material.

Date	08.08.	13.08.
Material	CSF	Intraoperative material
Erythrocytes (Mpt/L)	0.3	Not tested
Leukocytes (Mpt/L)	211.9	Not tested
Neutrophils (%)	3	Not tested
Lymphocytes (%)	80	Not tested
Monocytes (%)	14	Not tested
Total protein (mg/L)	1601	Not tested
Albumin (mg/L)	947	Not tested
Q albumin (CSF/serum)	38.8	Not tested
Q IgG (CSF/serum)	36.6	Not tested
Q IgA (CSF/serum)	24.7	Not tested
Q IgM (CSF/serum)	9.2	Not tested
Lactate (mmol/L)	3.87	Not tested
Glucose (mmol/L)	5.43	Not tested
Q glucose (CSF/Serum)	0.39	Not tested
*C. neoformans* PCR	Negative	Negative
*C. neoformans* antigen	Not tested	Not tested
*C. neoformans* culture	Not tested	Negative
*C. neoformans* microscopy	Not tested	Not tested

## Data Availability

No underlying data were collected or produced in this study.
